# Quality Improvement Project to Improve GP Referrals to a Rural Psychiatric Team

**DOI:** 10.1192/bjo.2022.344

**Published:** 2022-06-20

**Authors:** George Tresilian, Hamnah Nasser, Su Hyun Park, Dehneez Asad, Raja Akbar Khan, Helen Moran, Vishnu Gopal

**Affiliations:** 1 University of Sheffield, Sheffield, United Kingdom; 2 Nottingham Healthcare NHS Foundation Trust, Nottingham, United Kingdom; 3 Derbyshire Healthcare NHS Foundation Trust, Derby, United Kingdom

## Abstract

**Aims:**

With increasing awareness and reduction of stigma associated with Mental Health issues, referrals to services are increased, pushing specificity of commissioning and therefore declining patients of services when referrals are inadequate. Standards would be improved by better inclusion of information necessary for the Single Point of Access process (SPOA) in the Bolsover Community Mental Health Team (CMHT) to make prompt, effective decisions on allocating care.

A Quality Improvement project in a Mental Health Team was devised to improve standards, and acceptance rate, of appropriate referrals to the Bolsover CMHT from General Practitioners (GPs). This would encourage GPs to refer patients whose mental health difficulties do not meet CMHT thresholds to alternative services. A higher acceptance rate and lower rejection rate would indicate that the proportion of suitable referrals had increased.

**Methods:**

Using the Plan, Do, Study, Act (PDSA) model, Driver diagrams were used to create a template with the crucial information necessary for GP referrals to psychiatry/SPOA. Data were collected to check aims of the referral, sufficient information of the presenting complaint, personal & family history, safety concerns, protective factors, comorbidities, medication and substance misuse. The outcome of each referral was recorded and categorised as either Community Psychiatric Nurse Assessment, Outpatient Appointment, Referral Rejected, Referred Elsewhere or No Patient Response.

All referrals in September and October 2021 were analysed to assess whether enough information had been included for each variable. The September and October data were compared to check if the template had been associated with improved quality of referrals.

**Results:**

Pre-template, 17.4% of referrals were accepted, 13.0% received a SPOA assessment, 17.4% were rejected, 39.1% were re-referred elsewhere and 21.8% did not respond to the CMHT. After the template was circulated, 28.0% were accepted, 36.0% received a SPOA assessment, 4% received joint Doctor-SPOA care, 8% had a medication review and 12% were waiting for an MDT decision when data were analysed. The results for SPOA assessment and rejection were statistically significant (p < 0.05), while results for other outcomes were not.

Information on presenting complaint (82.1% to 100%, p < 0.05), personal history (39.3% to 92.3%, p < 0.05) and aims (50% to 88%, p < 0.05) increased, while other information did not change in a statistically significant manner.

**Conclusion:**

The template led to an increased proportion of accepted referrals and a decreased proportion of rejected referrals. However, information on variables did not necessarily improve in the same manner. The template is useful to improve decision-making in SPOA.

